# Benign meningioma manifesting with acute subdural hematoma and cerebral edema: a case report and review of the literature

**DOI:** 10.1186/s13256-021-02935-x

**Published:** 2021-06-29

**Authors:** Ji Won Nam, Eun Suk Park, Jun Bum Park, Jae Hee Seo, Minsoo Kim, Na Young Jung

**Affiliations:** 1 Department of Neurosurgery, Ulsan University Hospital, University of Ulsan College of Medicine, Ulsan, Republic of Korea; 2 Department of Pathology, Ulsan University Hospital, University of Ulsan College of Medicine, Ulsan, Republic of Korea; 3 Department of Neurosurgery, Gangneung Asan Hospital, Gangneung, Republic of Korea

**Keywords:** Subdural hematoma, Brain neoplasm, Meningioma, Brain edema

## Abstract

**Background:**

Spontaneous subdural hematoma rarely presents with a hypervascular or malignant tumor but even less frequently in a benign tumor like meningioma. We encountered a patient with acute subdural hematoma associated with benign meningioma. Here, we report this case along with a review of previous reports, especially focusing on their clinical features and possible bleeding mechanisms.

**Case presentation:**

A 53-year-old Asian woman presented with severe headache and progressive neurologic deterioration due to cerebral edema. The patient was submitted to open surgery for evacuation of the subdural hematoma and concurrent tumor removal on the ipsilateral parietal convexity. A hypervascular, encapsulated mass was identified during surgery and completely removed including the adjacent dura mater (Simpson grade 0). The tumor was histologically confirmed as an angiomatous meningioma (World Health Organization grade I). Her clinical course was uneventful after surgery.

**Conclusions:**

Although meningiomas are commonly benign according to their histological traits, they can lead to spontaneous bleeding and cause neurologically unstable condition. Therefore, meningiomas need to be considered as a cause of spontaneous subdural hematoma if radiologically suspicious, which should be reflected by proper management for a positive outcome.

**Supplementary Information:**

The online version contains supplementary material available at https://doi.org/10.1186/s13256-021-02935-x.

## Background

Meningioma is one of the most frequently reported intracranial tumors. It accounts for 37.6% of all primary brain tumors and 53.3% of nonmalignant tumors [1]. Although the majority of meningioma cases are incidentally identified, they commonly present with headache, dizziness, seizures, or focal neurological deficits showing variability in clinical features depending on tumor size, location, invasion of adjacent brain tissues or cerebral vessels, and obstruction of cerebrospinal fluid pathways [2]. About 80–90% of meningioma cases are classified as World Health Organization (WHO) grade I, known as histopathologically benign tumors [1, 3]. Despite their benign features with a lower risk of bleeding than malignant tumors, they can bleed as a result of radiation therapy, preoperative embolization, or spontaneous events with an incidence of 1.3–2.4% [4, 5]. The most common form of bleeding related to meningioma is the subarachnoid hemorrhage, followed by intracerebral hemorrhage [6, 7]. In particular, hemorrhages present less frequently as a subdural hematoma (SDH), with incidence reportedly less than a quarter that of intracranial hemorrhage in patients with meningioma, although, paradoxically, the typical meningioma location is the subdural space [5, 8, 9].

Here, we report the case of a middle-aged woman with nontraumatic SDH and cerebral edema originating from a small convexity meningioma. In the wake of this case, we discuss the possible mechanisms of tumor bleeding, as well as the outlines of this condition, with a review of the previously published literature.

## Case presentation

**Fig. 1:**
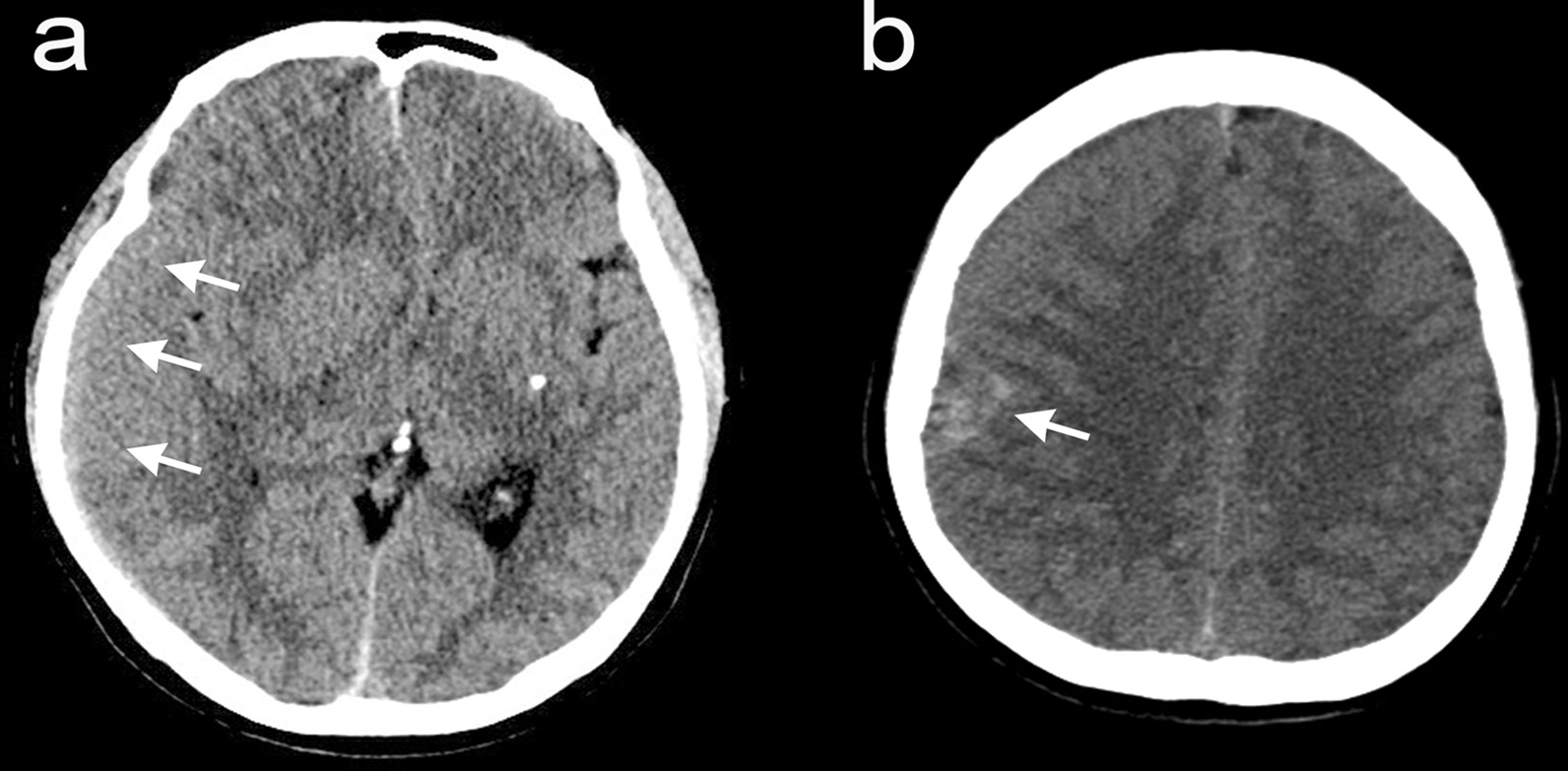
Preoperative axial computed tomography showing an acute subdural hematoma extending to the right fronto-temporo-parietal area (**a**, arrows) and a round-shaped, high-density mass on the right parietal convexity (**b**, arrow).

**Fig. 2:**
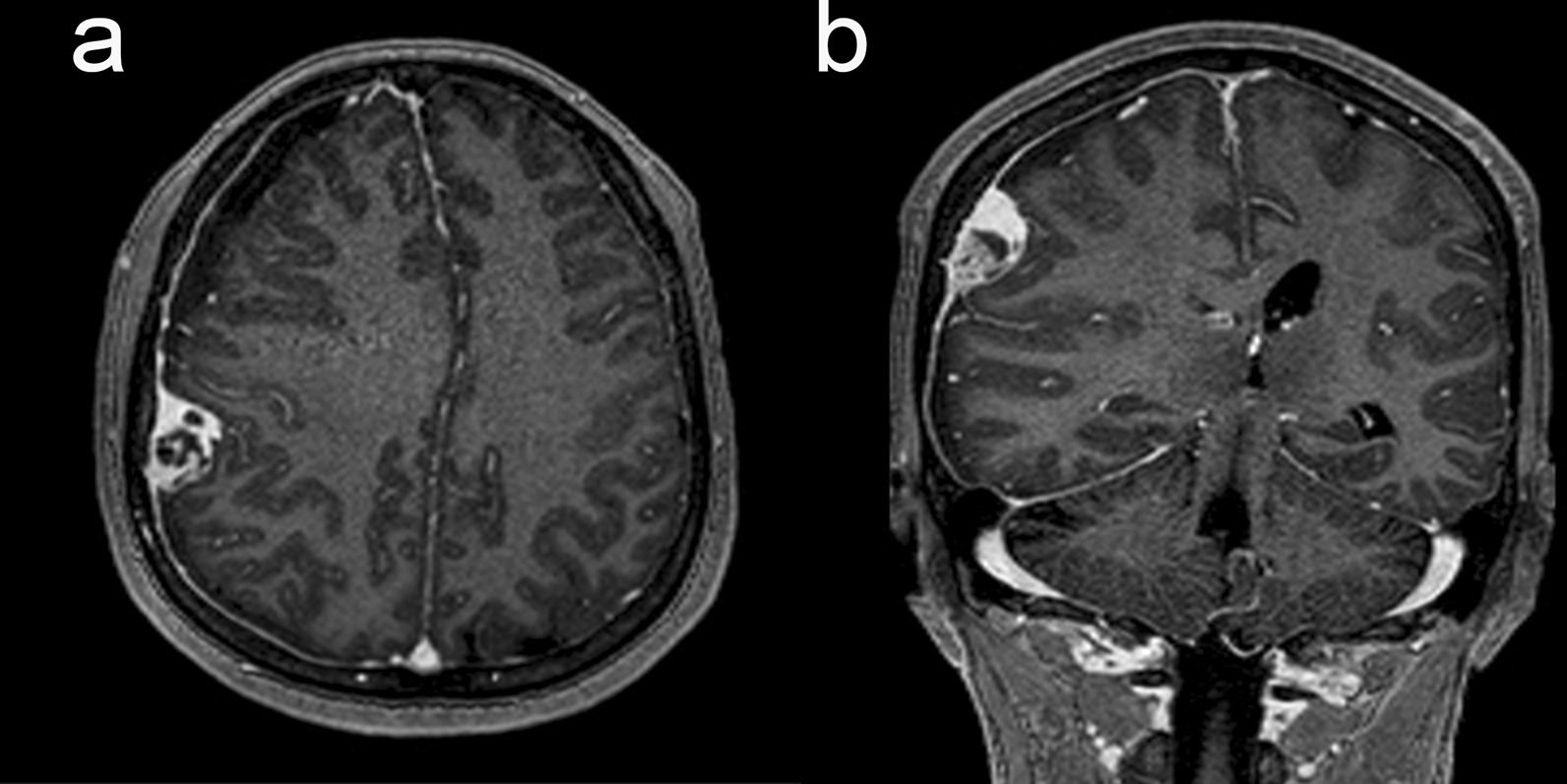
T1-weighted brain magnetic resonance images demonstrating a heterogeneously enhanced, 2.6 × 1.5 cm sized, extraaxial mass on the right parietal convexity with diffuse subdural hematoma in both axial view (**a**) and coronal view (**b**)

A 53-year-old Asian woman visited our emergency department complaining of progressive headache and vomiting for 2 days. There was no traumatic event related to her symptoms. She had no previous medical history, and all laboratory tests were within the normal range. Her vital signs were normal. Initial neurologic examination showed alert consciousness and no abnormal neurologic signs. Brain computed tomography (CT) scan revealed a right-sided acute-type SDH with a focal, round-shaped, high-density mass in the right parietal convexity (Fig. 1). Brain magnetic resonance imaging (MRI) demonstrated a heterogeneously enhanced, round-shaped, 2.6 × 1.5 cm sized, extraaxial mass along with diffuse SDH in the right fronto-temporo-parietal area, as well as mild midline shifting (Fig. 2). She was admitted to the neurosurgical department, then medically managed for increased intracranial pressure with intravenous mannitol and steroid administration. On the next day, her neurologic status progressively deteriorated, resulting in stupor. Follow-up CT scan revealed an uncal herniation with no increase in SDH volume.

**Fig. 3:**
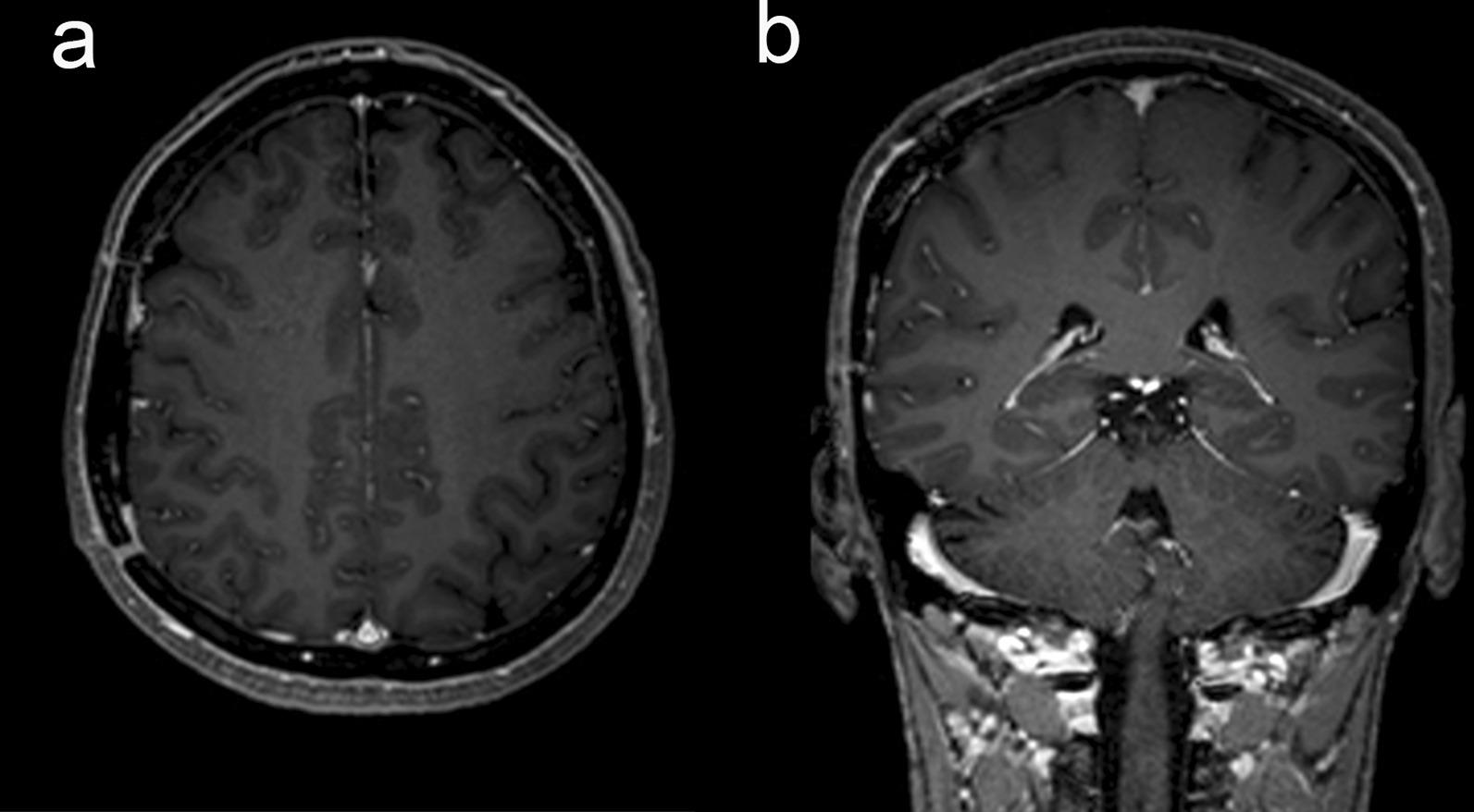
Six-month follow-up brain magnetic resonance images revealing no residual meningioma or subdural hematoma

She underwent an emergency operation. After craniotomy and dura opening, an acute type SDH was noted. The mass lesion identified on MRI was not clearly visible because of thick blood clots. When the dura mater was reflected, the bloody mass lesion inside the dura mater was clearly detached from the cortex without any dissection. This mass measured 1.5 cm in maximal diameter and was covered with fresh blood clots. There was only a small tearing on a cortical vein adhered to the tumor base arachnoid capsule. After gross total removal of the mass and its surrounding dura extending 2 cm from the tumor margin (Simpson grade 0), bleeding was controlled. There was no other active bleeding point, such as a cortical artery or bridging vein in the subdural space. Brain swelling was not excessive, the bone flap was closed, and the wound was approximated layer by layer. In the postoperative course, her neurologic deterioration reverted to normal right after surgery. She was discharged on the 10th postoperative day without any focal neurologic deficit. The follow-up brain MRI taken 6 months later showed no tumor or hemorrhage relapse (Fig. 3).

**Fig. 4:**
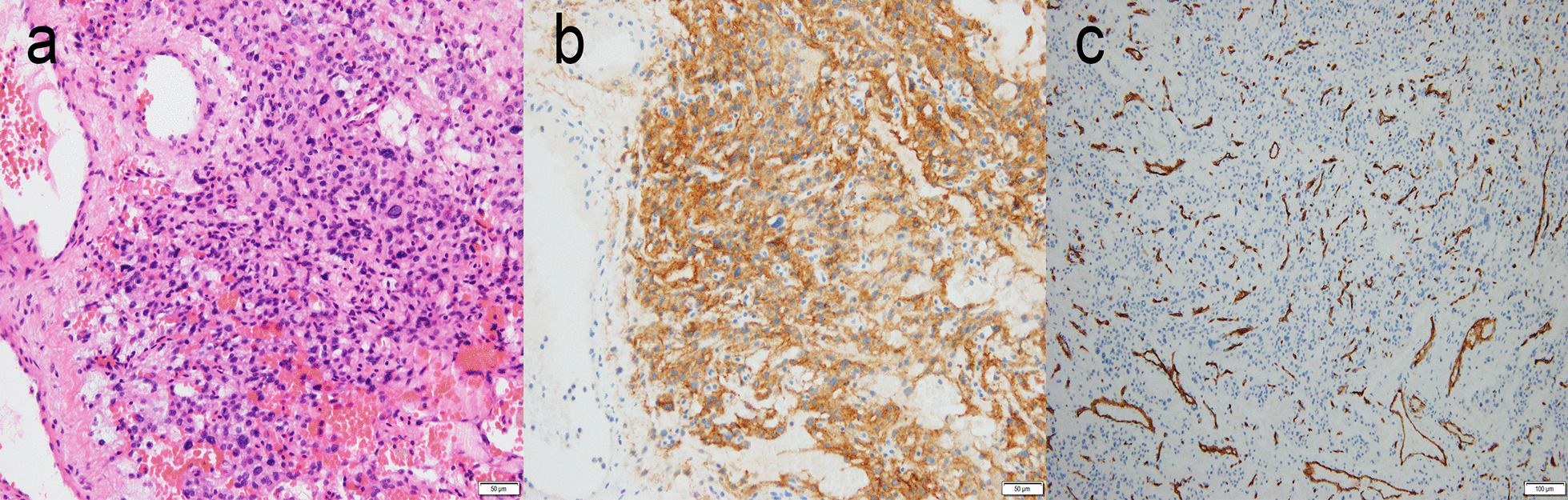
Microscopically, the tumor consists of predominant blood vessels with markedly hyalinized vascular walls (**a**: hematoxylin–eosin, ×200) and shows positive immunoreactivity for epithelial membrane antigen (EMA) (**b**: EMA, ×200) and CD34 (**c**: CD34, ×100), all contributing to the histopathologic diagnosis of an angiomatous meningioma of WHO grade I

Histopathologic examination of the tumor revealed hypercellular components with numerous vascular structures. Mitosis was rare (≤ 2 per ten high-power fields), and the proliferation index (Ki-67) was low, with 3%. No microscopic invasion of the dura or brain tissue was identified, as well as no evidence of malignancy, necrosis, atypia, or anaplasia (Fig. 4). These characteristics supported the histopathologic diagnosis of angiomatous meningioma, a benign meningioma of WHO grade I.

## Discussions

Spontaneous SDH is defined as any unusual disruption of cranial vessels within the subdural space without traumatic events [10]. It is less reported compared with traumatic SDH. Major causes include rupture of cortical arteries, coagulopathy, vascular malformation or aneurysm, neoplasm, cocaine use, or spontaneous intracranial hypotension [11]. Among them, 5.4% of spontaneous SDH cases are associated with brain tumors, especially in middle-aged adults like in our case [11, 12]. Metastatic brain tumors or high-grade primary tumors are prone to intracranial hemorrhage because of their malignant histological characteristics and common association with coagulopathy [13]. However, even benign tumors like meningioma or schwannoma can present with bleeding [4].

Hemorrhagic meningiomas are reported in 1.3–2.4% of all meningioma cases [4, 5]. The bleeding tendency of meningioma is known to be increased in convexity and intraventricular locations, as well as in fibroblastic, malignant, or angioblastic subtypes [8, 9, 14, 15]. Matsuoka *et al*. reviewed nine cases of repeated bleeding from benign meningiomas and demonstrated that its related mortality of 28–50% is not negligible [16]. Therefore, in such a case, immediate diagnosis and surgical treatment by hematoma evacuation with underlying tumor resection should be performed.

SDH is a rare type of hemorrhage related to meningioma, particularly in cases with benign histology. Table 1 summarizes the 66 reported cases of histologically confirmed benign meningioma (WHO grade I) presenting with SDH, including the present case [6, 7, 16–27] . Detailed information is listed in Additional file 1. The mean age is 61.9 years (range, 26–85 years), and the reported cases are predominantly female (male:female = 28:38). Among them, a convexity location has a higher probability of an association with SDH, accounting for about 74.2%, similar to the 80% in other studies [15, 25], although some authors still hold that the tumor location has no correlation with hemorrhagic events [17]. In terms of the histological meningioma types, reviews of previously reported cases show that the risk of spontaneous SDH is highest in the meningothelial type, followed by the transitional type [25, 26, 28]. According to Table 1, it is evident that SDH frequently occurs in the meningothelial type (26 cases, 39.4%), followed by angiomatous and transitional types (11 cases each, 16.7%). Meningothelial meningioma is one of the most common histology in benign meningiomas; its incidence of SDH is also high. In addition, hemorrhage in the meningothelial subtype is probably related to the presence of vasoactive substances released by the tumor, which could induce vasodilatation and resultant bleeding [18]. In the angiomatous meningioma subtype that includes our case, only a little over ten cases have been reported so far.

**Table 1:** Characteristics of World Health Organization (WHO) grade I meningiomas presenting with subdural hematoma

Number of patients	66
Male/female	28/38
Mean age	61.9 years (range 26–85 years)
Location	
Convexity	49 (74.2%)
Falcine	5
Sphenoidal	5
Parasagittal	4
Skull base/tentorial/posterior fossa	1/1/1
Histology	
Meningothelial	26
Angiomatous	11
Transitional	11
Fibrous	8
Not further specified	8
Microcystic/mixed	1/1
Outcome	
Alive	47
Alive with deficit	7
Dead	5
Not available	7

Angiomatous meningioma is a rare subtype of meningioma. The tumor is characterized by highly vascularized tissues intervening in the background areas of the syncytial pattern of typical meningioma cells [29, 30]. Its vascular channels mostly consist of small- or medium-sized, markedly hyalinized walls [31, 32]. Its bleeding tendency is due to its numerous vascular structures and the frequently accompanying peritumoral edema at disease onset, possibly related to a significant role of the angiogenic protein vascular endothelial growth factor. Hua *et al*. noted that peritumoral edema is encountered in 47.1–100.0% of angiomatous meningioma in their review [32]. This may explain the progressive cerebral edema and rapid clinical deterioration of the present case. Fortunately, the clinical outcome of this tumor is generally benign, like in our case, due to the grade I histologic characteristics.

The mechanism of SDH associated with benign meningioma is still obscure. Previous reports suggested several hypothetic mechanisms based on intraoperative findings or pathologic examinations. They include (1) rupture of a weak point of the vessel wall in the distended feeding artery with tumor growth; (2) disruption of abnormally developed vessels in the tumor, causing intratumoral hemorrhage or necrosis; (3) histochemical changes by vasoactive substances released by the tumor itself, consequently leading to neovascularization or forming a neomembrane such as in chronic SDH; and (4) potential friability of the bridging vessels or subdural veins stretched by the tumor mass and, thus, susceptibility to minor trauma [6, 7, 15]. In our case, we identified no cellular necrosis inside the tumor but only hypervascular structures and related bleeding. Instead, a small tearing on the cortical vein was revealed right beneath the tumor, suggesting mechanical stretching and distortion of bridging vessels by inertial effects of the tumor mass. In previous cases, some authors also demonstrated similar findings related to preceding minor trauma caused by events such as vigorous coughing or a Valsalva maneuver [12, 15, 33]. However, most of the bleeding meningiomas were not explained by only one of the several pathophysiologic conditions but a combination of various possible factors.

According to Table 1, patients with meningioma and SDH generally recovered completely with no residual abnormalities, although rarely some neurological symptoms remained after surgery. However, about 10% of the patients were left with neurological sequelae, and 7.5% (five patients) died despite proper management. Therefore, an optimistic patient prognosis is not indicated just based on the histopathologic classification as a benign tumor.

## Conclusion

Benign convexity meningiomas rarely develop an SDH, and their clinical progress can be worse than expected. Therefore, a meningioma should be fully suspected as the cause of a spontaneous SDH in cases with no vascular abnormalities or coagulopathies. In addition, early diagnosis and proper management including hematoma evacuation and tumor removal are recommended to alleviate a patient’s neurologic condition and to improve the prognosis.

## Supplementary Information

** Additional file 1.** Detailed characteristics of World Health Organization (WHO) grade I meningiomas presenting with subdural hematoma.

## Data Availability

All data generated or analyzed during this study are included in this published article and its supplementary information file.

## Abbreviations

CT: Computed tomography
MRI: Magnetic resonance imaging
SDH: Subdural hematoma
WHO: World Health Organization
