# Supplementary material for: Benign meningioma manifesting with acute subdural hematoma and cerebral edema: a case report and review of the literature
Source: J Med Case Rep. 2021 Jun 29;15:335. doi: 10.1186/s13256-021-02935-x (PMC8244191; doi:10.1186/s13256-021-02935-x)
Supplement: Supplementary file 1 — Additional file 1. Detailed characteristics of World Health Organization (WHO) grade I meningiomas presenting with subdural hematoma. [file 13256_2021_2935_MOESM1_ESM.docx]

**Additional file 1.** Detailed characteristics of World Health Organization (WHO) grade I meningiomas presenting with subdural hematoma.

| No. | Author | Year | Age/Sex | Tumor location | Histology | Outcome |
| --- | --- | --- | --- | --- | --- | --- |
| 1 | Bingas et al. | 1966 | 65/F | Convexity | Meningothelial | Alive |
| 2 | Modesti et al. | 1976 | 49/F | Parasagittal | Meningothelial | Alive with deficit |
| 3 | Modesti et al. | 1976 | 69/M | Convexity | Meningothelial | Alive |
| 4 | Modesti et al. | 1976 | 59/M | Sphenoidal | Meningothelial | Alive |
| 5 | Modesti et al. | 1976 | 72/F | Parasagittal | Angiomatous | Dead |
| 6 | Walsh et al. | 1977 | 77/F | Convexity | Meningothelial | Dead |
| 7 | Everett et al. | 1979 | 65/M | Convexity | Meningothelial | NA |
| 8 | Reverdin et al. | 1981 | 49/F | Convexity | Angiomatous | Alive |
| 9 | Patil et al. | 1982 | 75/M | Convexity | Fibrous | Alive |
| 10 | Baskinis et al. | 1984 | 68/M | Convexity | Angiomatous | Alive |
| 11 | Tomita et al. | 1985 | 61/F | Convexity | Meningothelial | Alive |
| 12 | Kotwica et al. | 1986 | 32/M | Convexity | Angiomatous | NA |
| 13 | Itoyama et al. | 1987 | 63/F | Sphenoidal | Transitional | Alive |
| 14 | Tokunaga et al. | 1988 | 61/F | Convexity | Transitional | Alive |
| 15 | Jones et al. | 1989 | 76/F | Skull base | Meningothelial | Dead |
| 16 | Takahashi et al. | 1989 | 46/F | Posterior fossa | Fibrous | Alive |
| 17 | Niikawa et al. | 1990 | 49/F | Convexity | Meningothelial | Alive |
| 18 | Chang et al. | 1990 | 56/F | Convexity | Meningothelial | Alive |
| 19 | Martinez-Lage et al. | 1991 | 70/M | Convexity | Meningothelial | NA |
| 20 | Renowden et al. | 1992 | 41/F | Parasagittal | Meningothelial | Alive |
| 21 | Chaskis et al. | 1992 | 59/F | Convexity | Angiomatous | Alive |
| 22 | Chaskis et al. | 1992 | 62/M | Convexity | Meningothelial | Dead |
| 23 | Chen et al. | 1992 | 79/M | Convexity | Meningothelial | Alive |
| 24 | Russell et al. | 1993 | 55F | Convexity | Transitional | Alive |
| 25 | Ueno et al. | 1993 | 67/M | Convexity | Meningothelial | Alive |
| 26 | Pozzi et al. | 1993 | 73/F | Convexity | Transitional | Alive |
| 27 | Pozzi et al. | 1993 | 85/F | Convexity | NS | Alive |
| 28 | Koumtchev et al. | 1993 | 26/F | Convexity | Fibrous | Alive with deficit |
| 29 | Popovic et al. | 1994 | 47/F | Convexity | Meningothelial | Alive |
| 30 | Tanaka et al. | 1994 | 47/F | Convexity | Meningothelial | Alive |
| 31 | Spektor et al. | 1995 | 73/F | Convexity | Angiomatous | Alive with deficit |
| 32 | Scarrow et al. | 1998 | 77/F | Convexity | Fibrous | Alive |
| 33 | Sunada et al. | 1998 | 48/F | Convexity | Fibrous | Alive with deficit |
| 34 | Shimizu et al. | 1998 | 67/M | Convexity | Meningothelial | NA |
| 35 | Moriyama et al. | 1998 | 68/M | Convexity | Angiomatous | NA |
| 36 | Timothy et al. | 1999 | 64/M | Convexity | Transitional | Alive |
| 37 | Okuno et al. | 1999 | 78/F | Falcine | Transitional | Alive with deficit |
| 38 | Sinha et al. | 2001 | 68/M | Convexity | NS | Alive |
| 39 | Sinha et al. | 2001 | 70/F | Convexity | NS | Alive |
| 40 | Lefranc et al. | 2001 | 62/M | Convexity | Meningothelial | Dead |
| 41 | Lefranc et al. | 2001 | 68/F | Convexity | Transitional | Alive |
| 42 | Lefranc et al. | 2001 | 59/F | Convexity | Angiomatous | Alive |
| 43 | Bruno et al. | 2003 | 77/M | Convexity | Transitional | Alive |
| 44 | Goyal et al. | 2003 | 66/M | Falcine | Transitional | Alive |
| 45 | De Silva et al. | 2004 | 61/F | Sphenoidal | Meningothelial | NA |
| 46 | Di Rocco et al. | 2006 | 72/M | Convexity | Meningothelial | Alive |
| 47 | Di Rocco et al. | 2006 | 74/M | Convexity | Transitional | Alive |
| 48 | Mitsuhara et al. | 2006 | 60/F | Tentorial | Meningothelial | Alive |
| 49 | Kashimura et al. | 2008 | 55/M | Convexity | Meningothelial | Alive |
| 50 | Worm et al. | 2009 | 64/M | Falcine | NS | Alive with deficit |
| 51 | Lakshmi Prasad et al. | 2010 | 73/M | Sphenoidal | NS | Alive |
| 52 | Deprez et al. | 2012 | 66/M | Convexity | NS | Alive with deficit |
| 53 | Chonan et al. | 2013 | 67/F | Convexity | Meningothelial | Alive |
| 54 | Rocha et al. | 2013 | 52/M | Convexity | NS | NA |
| 55 | Hambra et al. | 2014 | 59/M | Sphenoidal | Angiomatous | Alive |
| 56 | Levine et al. | 2014 | 69/M | Convexity | NS | Alive |
| 57 | Eljebbouri et al. | 2014 | 51/M | Convexity | Meningothelial | Alive |
| 58 | Kim et al. | 2015 | 61/F | Convexity | Fibrous | Alive |
| 59 | Krishnan et al. | 2015 | 62/M | Parasagittal | Fibrous | Alive |
| 60 | Suzuki et al. | 2018 | 61/F | Falcine | Angiomatous | Alive |
| 61 | Sadegh Masoudi et al. | 2019 | 36/F | Convexity | Fibrous | Alive |
| 62 | Aloraidi et al. | 2019 | 49/F | Convexity | Angiomatous and microcystic | Alive |
| 63 | Aloraidi et al. | 2019 | 49/F | Convexity | Meningothelial | Alive |
| 64 | Matsuoka et al. | 2019 | 61/F | Falcine | Transitional | Alive |
| 65 | Nery et al. | 2019 | 85/F | Convexity | Microcystic | Alive |
| 66 | Present case | 2020 | 53/F | Convexity | Angiomatous | Alive |

M: male, F: female, NA: not available, NS: not further specified, just grade I histology
